# Blood pressure and adverse cardiovascular outcomes in older people with type 2 diabetes and chronic kidney disease: Findings based on the Clinical Practice Research Datalink databases in England

**DOI:** 10.1111/dom.70234

**Published:** 2025-10-28

**Authors:** Anna Meffen, Freya Tyrer, Junior Mbah Mbahnjeck, Kamlesh Khunti, Francesco Zaccardi, Setor K. Kunutsor

**Affiliations:** ^1^ Leicester Real World Evidence Unit, Diabetes Research Centre University of Leicester Leicester UK; ^2^ Faculty of Medicine Imperial College London London UK; ^3^ Section of Cardiology, Department of Internal Medicine, Max Rady College of Medicine, Rady Faculty of Health Sciences University of Manitoba Winnipeg Manitoba Canada

**Keywords:** Blood pressure, cardiovascular disease, Chronic kidney disease, heart failure, Major adverse cardiovascular events, primary care, real‐world evidence, type 2 diabetes

## Abstract

**Aims:**

Managing blood pressure (BP) in older adults with type 2 diabetes (T2D) and chronic kidney disease (CKD) remains controversial, particularly regarding optimal targets and the impact of inadequate monitoring. Using Clinical Practice Research Datalink (CPRD) data, this study assessed associations between baseline systolic and diastolic BP (SBP and DBP) and the risk of major adverse cardiovascular events (MACE) and mortality in adults aged ≥65 with both T2D and CKD, the impact of missing BP records (proxy for inadequate monitoring), and sex‐based differences in these relationships.

**Materials and Methods:**

A retrospective cohort study was conducted using CPRD data. The primary outcome was MACE (nonfatal stroke, myocardial infarction, and cardiovascular death); the secondary outcome was all‐cause mortality. Baseline BP was modelled continuously and categorised as high (≥140/90 mmHg), normal (<140/90 mmHg), or missing (no BP record within 2 years prior to diagnosis). Flexible parametric competing risks models estimated adjusted 5‐year outcome risks.

**Results:**

MACE analysis included 160 764 individuals; mortality analysis included 181 307. The 5‐year MACE risk was 14.1% for normal, 13.8% for high, and 19.6% for missing SBP. For all‐cause mortality, risks were 20.6% (normal), 19.4% (high), and 34.0% (missing). SBP was a stronger risk indicator than DBP for both outcomes. Lower SBP (120 mmHg) was moderately associated with increased MACE and mortality; higher DBP (90 mmHg) was linked to increased mortality. Men had higher MACE and mortality risks than women.

**Conclusions:**

In older adults with T2D and CKD, lower SBP and DBP were moderately associated with a higher risk of MACE and mortality, but the strongest indicator of adverse outcomes was the absence of regular blood pressure monitoring.

## 1 INTRODUCTION

Type 2 diabetes (T2D) poses a significant global health burden, contributing to substantial morbidity and mortality.^1^ Cardiovascular disease (CVD) remains the leading cause of death in individuals with T2D.^1^ People with T2D have a high prevalence of multiple long‐term conditions including hypertension and chronic kidney disease (CKD).^1^, ^2^ Effective management of key modifiable risk factors, including blood pressure (BP), is essential to reduce the progression of disease and risk of major adverse cardiovascular events (MACE).^3^ Hypertension, highly prevalent in individuals with T2D, is a well‐known risk factor for MACE and mortality.^4^, ^5^ Evidence from randomised controlled trials (RCTs) and observational studies has shown that BP control plays a critical role in reducing cardiovascular morbidity and mortality in people with T2D.^6^, ^7^, ^8^ However, determining optimal BP targets in older adults is challenging.^9^ Although more intensive BP lowering is associated with cardiovascular protection in some populations,^10^ this approach remains controversial in older individuals. Several studies have reported that stringent BP control in this group may lead to adverse outcomes, including increased mortality, falls, kidney dysfunction, and cognitive decline.^11^, ^12^ Moreover, most hypertension guidelines are based on RCTs that underrepresent or exclude older adults, particularly those with multimorbidity, thereby limiting their generalizability.^13^, ^14^

BP lowering in patients with CKD has also been associated with reduced CVD events and mortality.^15^ The coexistence of hypertension, T2D, and CKD is particularly common in older adults,^16^ yet the interplay of these conditions and the optimal BP targets for cardiovascular benefit in this population remain poorly understood. A major limitation in the current literature is the lack of high‐quality evidence derived from trials that include this high‐risk group.^17^ As such, real‐world data are essential to bridge this evidence gap and better characterise the association between BP levels and adverse cardiovascular outcomes in older adults with T2D and CKD.

In addition, while considerable attention has been placed on BP levels and treatment thresholds, less is known about the prognostic implications of missing BP records in routine care. Missing BP data may signal gaps in care, suboptimal monitoring, or disengagement from clinical follow‐up, all of which could confer elevated cardiovascular risk.^18^ The extent to which missing BP records influence clinical outcomes has not been adequately explored and remains an overlooked area of investigation.

Therefore, this study provides novel insights by using real‐world primary care data based on the Clinical Practice Research Datalink (CPRD) to simultaneously examine: (1) the association between baseline systolic and diastolic BP and the risk of MACE and all‐cause mortality in older adults with T2D and CKD; (2) the impact of missing BP records as a proxy for inadequate monitoring or healthcare engagement; and (3) potential sex‐based differences in these relationships. This work aims to inform more tailored and pragmatic BP management strategies in this high‐risk, underrepresented population.

## 2 MATERIALS AND METHODS

A research protocol was accepted by CPRD's Research Data Governance (RDG) Process (Protocol #23_002560).^19^ Analyses were conducted in accordance with the Reporting of Studies Conducted Using Observational Routinely‐collected Data (RECORD) guidelines.^20^

### 2.1 Cohort definition

The study used de‐duplicated data from Aurum and GOLD CPRD databases with available linkage to Hospital Episode Statistics (HES) and Office for National Statistics (ONS) death records.^21^

Inclusion criteria comprised adults aged 65 years or over with a dual diagnosis of T2D and CKD (stages 3 to 5) where the second of those diagnoses (i.e., T2D in patients with CKD or vice versa) occurred from 1 January 2000 at an up‐to‐standard GP surgery (using CPRD's quality criteria). Index date (cohort entry) was the date of second diagnosis (i.e., T2D or CKD). Individuals were followed up until the date of last surgery update, event (if MACE), death, transfer out of the GP surgery, or (build date—01/06/2023) whichever occurred first. See Supplement 1 at https://github.com/DrAnnaMeffen/BP-MACE-2025 for more details.

Two nested cohorts were created for: (i) MACE outcome, excluding individuals with previous non‐fatal stroke and/or non‐fatal myocardial infarction (MI); and (ii) all‐cause mortality (all eligible individuals).

### 2.2 Exposure, outcomes and covariates

The main exposures of interest were systolic (SBP) and diastolic (DBP) blood pressure as both continuous and categorical measures. Baseline measurements were considered as those recorded nearest to the index date in the 2‐year window prior to diagnoses of CKD and T2D. To allow investigation of the impact of poor BP monitoring on adverse cardiovascular outcomes, both SBP and DBP were separately categorised into ‘high’ (≥140 mmHg SBP, ≥90 mmHg DBP), ‘normal’ (<140 mmHg SBP, <90 mmHg DBP) or categorised as ‘missing’ for those with no BP recording in the 2 years prior to the second diagnosis. The rationale for selecting the 140 mmHg threshold for systolic BP and 90 mmHg for diastolic BP as categorical cut‐offs is based on their widespread use across international hypertension guidelines^22^ as clinical treatment targets for initiating or intensifying antihypertensive therapy. These thresholds also reflect long‐standing convention in both clinical practice and observational research, facilitating comparability and interpretability of findings. We also selected SBP (120 and 140 mmHg) and DBP (70 and 90 mmHg) thresholds for risk comparison in the continuous analyses. These cut‐offs were chosen because they represent the lower and upper bounds commonly recommended in clinical guidelines. Specifically, SBP of 120 mmHg and DBP of 70 mmHg are frequently cited as intensive treatment targets,^15^, ^23^ whereas SBP of 140 mmHg and DBP of 90 mmHg reflect more conventional or conservative thresholds used in older adults or those with comorbidities such as T2D and CKD.^22^, ^24^ The two primary cardiovascular outcomes were 3‐point MACE—defined as hospitalisation for nonfatal stroke (HES), hospitalisation for nonfatal myocardial infarction (HES), and cardiovascular death (ONS deaths data)—and all‐cause mortality (ONS deaths data). Further details are in Supplement 1 at https://github.com/DrAnnaMeffen/BP-MACE-2025.

Covariates were chosen based on their known role as risk factors for cardiovascular disease. These were: age (years); ethnicity; alcohol consumption; smoking status; body mass index (BMI); CKD stage; and baseline prescription of T2D and CVD medication. Please see Supplement 1 at https://github.com/DrAnnaMeffen/BP-MACE-2025 for further information. Where no medication record was found, patients were assumed not to be receiving this treatment. Where ethnicity, alcohol consumption, smoking status, and BMI were unknown, a ‘missing’ category was created as the use of imputation methods for characteristic and lifestyle data is ethically debated, and imputation assumptions of data being missing at random are expected to be violated.^25^, ^26^

### 2.3 Analysis methods

The relationship between baseline blood pressure and MACE was investigated using a time‐to‐event analysis using SBP and DBP as continuous measures (where recorded). To account for the competing event of death from non‐cardiovascular causes, a competing risk flexible Royston–Parmar parametric model was fitted, calculating a 5‐year risk of MACE, unadjusted and adjusted for potential confounders, and estimated separately for men and women (as the risk is expected to differ by sex). The relationship between missing BP data and risk of MACE was investigated using separate models with SBP/DBP categorised into ‘high’, ‘normal’, and ‘missing’. Flexible parametric models were chosen for these analyses over more traditional Cox proportional hazards models and Fine and Gray competing risks models as the assumptions of proportional hazards were likely to be violated by the time‐dependent nature of the relationship assessed. Furthermore, the Fine and Gray models are greatly computationally intensive and would not be computationally possible with such large data and the institutionally available computing systems.

The same analyses (without competing risks) were repeated to investigate the relationships between baseline BP and all‐cause mortality. Analyses were performed in Stata 18 using the stpm3 and standsurv commands. Models considered four degrees of freedom (knots placed at the 25th, 50th, and 75th centile) for baseline effects and two degrees of freedom (knots at the 50th centile) for time‐varying effects of SBP/DBP and age (to relax the proportionality assumption).^27^, ^28^, ^29^

Two sensitivity analyses were conducted. The first investigated the relationship between baseline SBP and DBP with MACE and all‐cause mortality by CKD stage using the adjusted flexible parametric methods described above. While CKD stage is an important risk factor and indicator of disease severity, the two cohorts in this study predominantly had CKD Stage 3, meaning that it was necessary to group together those with CKD stage 4 and 5, thus limiting the ability of the study to accurately clarify this relationship; these results are therefore not included in the main manuscript. The second sensitivity analysis investigated the presumed violation of the proportional hazards assumption that led to the decision to employ flexible parametric methods rather than Cox Proportional Hazards methods. Cox models were fitted to the MACE and mortality cohort data and adjusted for all variables described above. These models were only completed for relationships involving continuous SBP, as the DBP and categorical relationships were expected to follow a similar pattern. Fine and Gray methods, which account for competing risks and assume proportional hazards of the subdistribution hazard, were too computationally intensive to compute on these large data. Results for the sensitivity analyses are described below with figures and tables in Supplement 2.

### 2.4 Patient and public involvement

Leicester Biomedical Research Centre has active Patient and Public Involvement groups to support existing research. This study is inspired by discussions where the importance of blood pressure recording within the general practice setting has been discussed.

## 3 RESULTS

### 3.1 Cohort characteristics

The study population comprised 181 307 individuals in the all‐cause mortality analyses. 160 764 (88.7%) without prior MACE were included in the MACE analysis (Table 1). Individuals who experienced MACE within the study period were more likely to be men, white, smokers/ex‐smokers, have normal BMI, have unknown alcohol consumption, CKD stage >3, and have lower SBP and DBP. They were less likely to be taking diabetes medication. The majority of the cohort were white (89.6%) and taking CVD medication (90.4%). Similar trends were observed for those in the mortality cohort within the study period.

**TABLE 1:** Cohort demographics by event and no event within the study period.

	MACE cohort		Mortality cohort	
	No MACE	MACE	No death	Death
*N*	137 504 (85.5%)	23 260 (14.5%)	137 454 (75.8%)	43 853 (24.2%)
Age (years)	77.0 (7.1)	79.9 (7.2)	76.5 (6.9)	80.6 (7.5)
Sex				
Men	60 885 (44.3%)	11 946 (51.4%)	62 892 (45.8%)	21 786 (49.7%)
Women	76 619 (55.7%)	11 314 (48.6%)	74 562 (54.2%)	22 067 (50.3%)
Ethnicity				
White	122 918 (89.4%)	21 159 (91.0%)	122 451 (89.1%)	40 261 (91.8%)
South Asian	5983 (4.4%)	868 (3.7%)	6642 (4.8%)	1286 (2.9%)
Black	3750 (2.7%)	463 (2.0%)	3800 (2.8%)	769 (1.8%)
Mixed	564 (0.4%)	60 (0.3%)	573 (0.4%)	106 (0.2%)
Other	2095 (1.5%)	235 (1.0%)	2140 (1.6%)	439 (1.0%)
Unknown	2194 (1.6%)	475 (2.0%)	1848 (1.3%)	992 (2.3%)
Smoking status				
Non‐smoker	67 289 (48.9%)	10 412 (44.8%)	66 770 (48.6%)	19 411 (44.3%)
Smoker	26 831 (19.5%)	4652 (20.0%)	27 058 (19.7%)	8843 (20.2%)
Ex‐smoker	43 215 (31.4%)	8152 (35.0%)	43 531 (31.7%)	15 424 (35.2%)
Unknown	169 (0.1%)	44 (0.2%)	95 (0.1%)	175 (0.4%)
BMI				
Underweight	1004 (0.7%)	251 (1.1%)	711 (0.5%)	725 (1.7%)
Normal weight	23 557 (17.1%)	4984 (21.4%)	22 097 (16.1%)	10 599 (24.2%)
Overweight	49 977 (36.3%)	8441 (36.3%)	50 852 (37.0%)	15 259 (34.8%)
Obese	61 415 (44.7%)	8996 (38.7%)	62 838 (45.7%)	15 593 (35.6%)
Unknown	1551 (1.1%)	588 (2.5%)	956 (0.7%)	1677 (3.8%)
Alcohol consumption				
Non‐drinker	22 385 (16.3%)	3762 (16.2%)	22 361 (16.3%)	7207 (16.4%)
Drinker	98 592 (71.7%)	15 639 (67.2%)	100 160 (72.9%)	28 194 (64.3%)
Ex‐drinker	1521 (1.1%)	309 (1.3%)	1509 (1.1%)	616 (1.4%)
Unknown	15 006 (10.9%)	3550 (15.3%)	13 424 (9.8%)	7836 (17.9%)
CKD stage				
3	131 550 (95.7%)	21 255 (91.4%)	132 269 (96.2%)	39 687 (90.5%)
4	5258 (3.8%)	1785 (7.7%)	4640 (3.4%)	3655 (8.3%)
5	696 (0.5%)	220 (0.9%)	545 (0.4%)	511 (1.2%)
Diabetes medication				
No record	66 936 (48.7%)	11 197 (48.1%)	66 883 (48.7%)	21 670 (49.4%)
Yes	70 568 (51.3%)	12 063 (51.9%)	70 571 (51.3%)	22 183 (50.6%)
CVD medication				
No record	13 648 (9.9%)	1837 (7.9%)	12 802 (9.3%)	4016 (9.2%)
Yes	123 856 (90.1%)	21 423 (92.1%)	124 652 (90.7%)	39 837 (90.8%)
Baseline SBP	137.5 (17.8)	136.2 (20.6)	137.7 (17.8)	135.6 (20.0)
Baseline DBP	74.3 (10.8)	73.6 (11.7)	74.3 (10.8)	73.9 (11.5)
Total sample size	MACE cohort = 160 764		Death cohort = 181 307	

*Note*: Mean(SD)/*n*(%). MACE cohort complete blood pressure data = 44 085 (27.4%), Mortality cohort complete BP data = 44 731 (24.7%).

Abbreviations: BMI, body mass index; DBP, diastolic blood pressure; MACE, major adverse cardiovascular events; SBP, systolic blood pressure.

### 3.2 MACE

**FIGURE 1:**
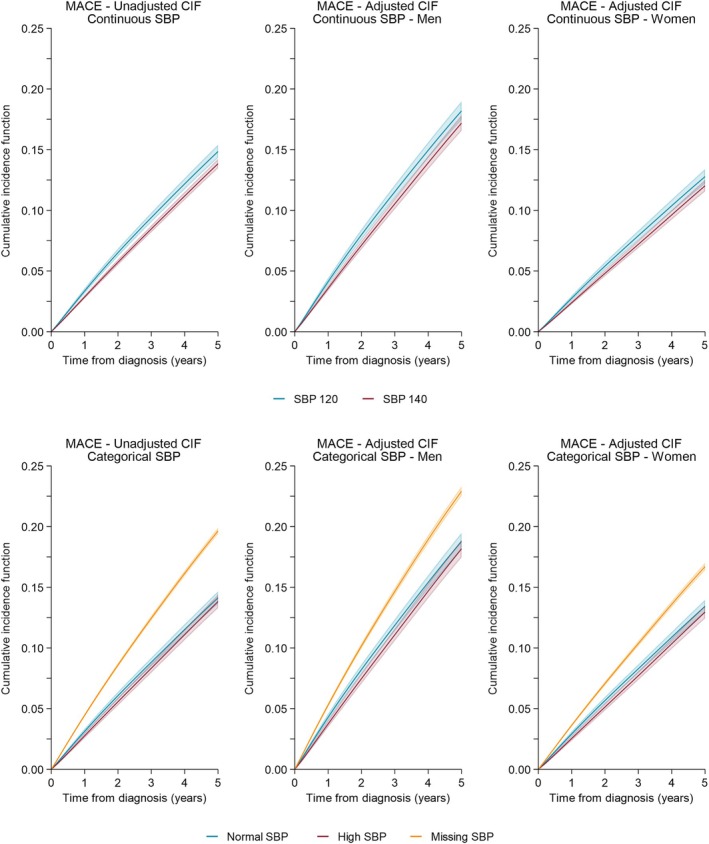
Cumulative incidence of MACE by systolic blood pressure—unadjusted and adjusted stratified for men and women. CIF, cumulative incidence function; MACE, major adverse cardiovascular events; SBP, systolic blood pressure.

**FIGURE 2:**
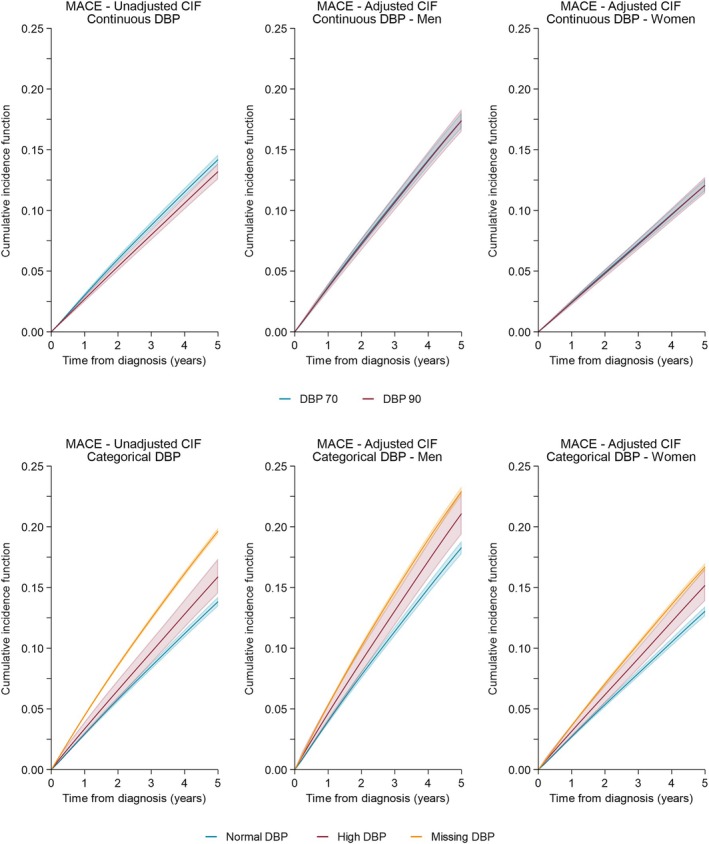
Cumulative incidence of MACE by diastolic blood pressure—unadjusted and adjusted stratified for men and women. CIF, cumulative incidence function; DBP, diastolic blood pressure; MACE, major adverse cardiovascular events.

Numbers at risk (for both cohorts) over the study period are presented by blood pressure category in Table 2. For those with complete data on continuous SBP (46 579 individuals), on average, the 5‐year unadjusted MACE risk was similar for those of SBP 120 compared to SBP 140—14.8% (95% CI: 14.3–15.4%) for SBP of 120 and 13.8% (95% CI: 13.5–14.2%) for SBP 140 (Figure 1). After adjustment, these trends remained with little difference between the two SBP levels; however, men had a higher risk—18.2% (95% CI: 17.4–19.0%) for men with SBP 120; 17.2% (95% CI: 16.6–17.8%) for men with SBP 140; 12.8% (95% CI: 12.2–13.4%) for women with SBP 120 and 12.0% (95% CI: 11.6–12.5%) for women with SBP 140. Similar trends were seen for DBP (Figure 2).

**TABLE 2:** Numbers at risk for MACE and mortality cohorts by year and SBP/DBP category.

Blood pressure category		Year 0	Year 1	Year 2	Year 3	Year 4	Year 5
MACE	SDP						
	Normal	24 005	20 518	17 235	14 312	11 729	9547
	High	20 080	17 468	14 837	12 475	10 418	8538
	Missing	116 679	98 592	82 081	67 752	55 492	45 052
	DBP						
	Normal	40 616	35 016	29 606	24 740	20 480	16 740
	High	3469	2970	2466	2047	1667	1345
	Missing	116 679	98 592	82 081	67 752	55 492	45 052
All‐cause mortality	SDP						
	Normal	24 342	20 972	17 757	14 859	12 252	10 054
	High	20 389	17 912	15 346	12 988	10 913	9014
	Missing	136 576	115 798	96 500	79 666	65 295	53 015
	DBP						
	Normal	41 188	35 810	30 533	25 690	21 399	17 633
	High	3543	3074	2570	2157	1766	1435
	Missing	136 576	115 798	96 500	79 666	65 295	53 015

Abbreviations: DBP, diastolic blood pressure; MACE, major adverse cardiovascular events; SBP, systolic blood pressure.

When considering the impact on MACE of having no blood pressure record (categorising blood pressure and including all 160764 individuals), the unadjusted MACE risk was 39% higher for missing SBP compared to normal SBP (14.1% (95% CI: 13.6–14.7%) for normal SBP, 19.6% (95% CI: 19.4–19.9%) for missing SBP, Figure 1). Similar trends were seen for men and women after adjustment for both SDP and DBP (Figures 1 and 2).

### 3.3 All‐cause death

**FIGURE 3:**
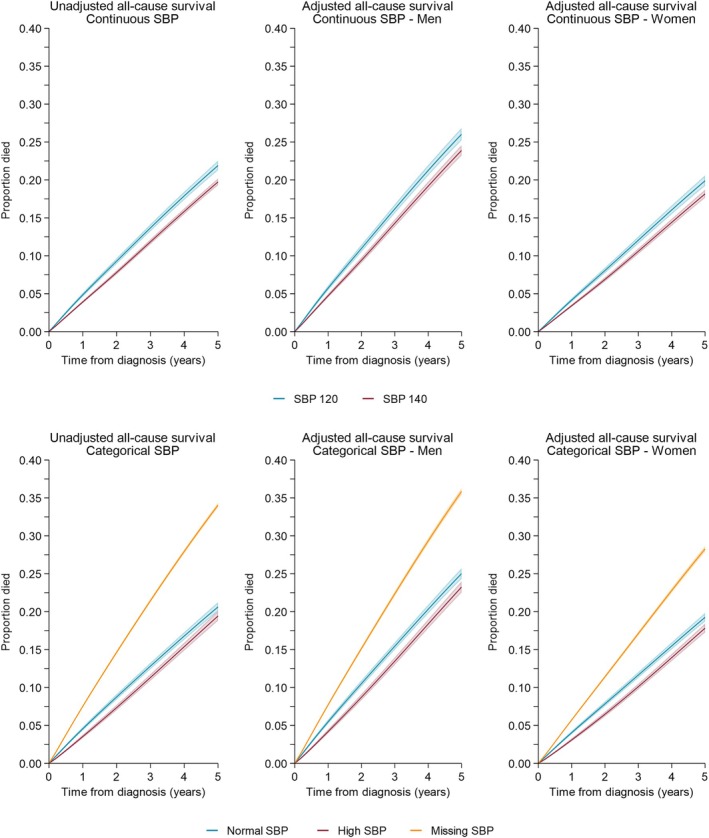
All‐cause survival proportion by systolic blood pressure—unadjusted and adjusted stratified for men and women. CIF, cumulative incidence function; SBP, systolic blood pressure.

**FIGURE 4:**
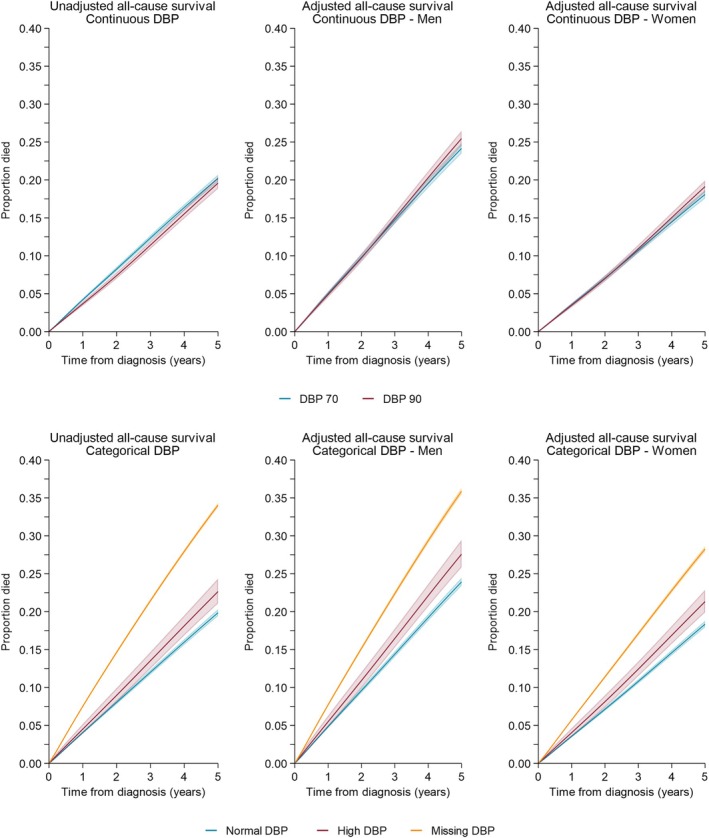
All‐cause survival proportion by diastolic blood pressure—unadjusted and adjusted stratified for men and women. CIF, cumulative incidence function; DBP, diastolic blood pressure.

For 47 374 individuals with complete SBP data, the unadjusted 5‐year mortality risk was slightly higher for those with SBP 120 compared with those with SBP 140; 21.9% (95% CI: 21.3–22.5%) for SBP 120 and 19.7% (95% CI: 19.3–20.2%) for SBP 140 (Figure 3). Similar trends were seen after adjustment, with risk higher for men than women—26.0% (95% CI: 25.2–26.8%) for men with SBP 120; 23.9% (95% CI: 23.2–24.6%) for SBP 140; 19.9% (95% CI: 19.2–20.6%) for women with SBP 120; 18.2% (95% CI: 17.6–18.7%) for women with SBP 140 (Figure 4). This slightly increased mortality risk was not seen in DBP analyses, where risks were similar for both DBP levels; however, risk remained higher for men.

When considering the impact on mortality risk of having no blood pressure record (including all 181 307 individuals), the unadjusted mortality risk was 65% higher for those with missing SBP compared to normal SBP (20.6% (95% CI: 20.0–21.2%) for normal SBP, 34.0% (95% CI: 33.8–34.3%) for missing SBP, Figure 3). Similar trends were seen for men and women after adjustment for both SDP and DBP (Figures 3 and 4).

### 3.4 Sensitivity analyses results

Sensitivity analyses performed to stratify MACE and mortality risk by CKD stage found similar patterns to the main analyses (Supplement 2, Section A). Specifically, those with missing SBP and DBP data had a higher risk of both MACE and mortality than those with complete BP data. Unsurprisingly, the risk of MACE and mortality was higher for those with CKD stages 4 and 5 by up to approximately two thirds compared to those with CKD Stage 3. However, due to a lack of data on those with CKD stages 4 and 5 and the necessity for combining these stages, these results should be interpreted with caution.

Sensitivity analyses performed to investigate the violation of the proportional hazard assumptions under Cox models found that the proportional hazards assumptions were indeed violated and the flexible parametric model choices in the main analyses were warranted (Supplement 2, Section B).

## 4 DISCUSSION

This study found that missing BP records within 2 years of diagnosis of T2D and CKD were associated with a substantially higher risk of MACE and all‐cause mortality compared to those with recorded high or normal BP measurements. Among those with recorded BP, lower SBP (120) was moderately linked to an increased risk of both MACE and mortality than higher SBP (140), while lower DBP (70) was moderately associated with a higher risk of MACE. Conversely, higher DBP (90) was moderately associated with an increased risk of mortality. Notably, there was little difference in MACE risk between those with high DBP and those with missing DBP records. Sex differences were observed, with men exhibiting slightly higher risks of MACE and mortality across BP comparisons. SBP was a stronger indicator of outcomes than DBP. The associations remained largely consistent after adjustment for covariates.

This study is the first to evaluate relationships between BP and MACE in older adults with T2D and CKD using real‐world data. While direct comparisons with prior studies are limited, BP and cardiovascular risk in these populations remain debated. Our findings indicate that lower SBP was moderately associated with an increased MACE and mortality risk, while lower DBP was linked to a higher MACE risk. Previous research reported conflicting evidence regarding optimal BP levels in older populations with diabetes or CKD. The Systolic Blood Pressure Intervention Trial demonstrated that in non‐frail older individuals with CKD and hypertension but without diabetes, targeting SBP of 120 compared to 140 reduced rates of major cardiovascular events and all‐cause mortality.^30^ Observational analyses of older populations have shown excess mortality associated with low SBP.^11^, ^31^, ^32^ In a cohort of older adults with CKD, without diabetes, SBPs higher than current targets did not increase mortality or cardiovascular outcomes, but SBPs <129 were associated with excess mortality.^33^ Among veterans with incident CKD, Kovesdy^34^ showed U‐shaped associations of SBP and DBP with mortality. A study by Hong^6^ found that cardiovascular events were more frequent in individuals with resting SBP ≥140 or DBP ≥95, but also in those with SBP <120 or DBP <65; the associations were consistent with U‐shaped patterns. Furthermore, a systematic meta‐analysis of 45 observational cohort studies comprising older patients with or without diabetes by Seidu^5^ revealed that SBP ≥140 was associated with increased cardiovascular outcomes, while SBP <120 was linked to a higher risk of all‐cause mortality.

Our findings support existing evidence that inadequate BP monitoring is associated with worse clinical outcomes.^17^ These findings highlight the importance of routine BP monitoring in older adults with T2D and CKD. Higher MACE and mortality risks in those with missing BP records suggest inadequate monitoring may reflect poor disease management or healthcare access barriers. The classification of missing BP may also capture broader issues such as low healthcare engagement, frailty, or other unmeasured factors, including cognitive impairment and social vulnerability. Although over 90% of individuals with missing BP records were receiving CVD medications at baseline, the absence of BP measurement within 2 years of diagnosis may reflect systemic deficiencies in risk factor assessment or disengagement from routine care. International guidelines recommend at least annual BP measurement for individuals with T2D and/or CKD, alongside the use of home or ambulatory BP monitoring to improve accuracy and engagement.^23^ Our findings support the need for policy and practice changes to ensure more consistent BP monitoring in high‐risk populations. This includes improving adherence to guideline‐recommended assessments, expanding home BP monitoring programmes, and implementing automated reminders or flags in electronic medical records to prompt annual BP checks. Targeted interventions to enhance primary care engagement and regular monitoring, particularly among frail, multimorbid, or socially vulnerable patients, may help identify those at highest risk and improve long‐term cardiovascular outcomes.

These results align with previous evidence indicating that lower BP targets in older adults with comorbidities, including T2D and CKD, may lead to adverse health outcomes.^11^, ^31^, ^32^, ^33^ This population is often frail and susceptible to the complications associated with intensive BP control. Several mechanisms may underlie the increased risk observed at very low BP levels (e.g., SBP 100–110 mmHg and DBP <70 mmHg), including compromised coronary and renal perfusion in the setting of advanced vascular ageing, autonomic dysfunction, and subclinical illness.^35^, ^36^, ^37^ Low BP in older multimorbid patients may also reflect overtreatment or reverse causality due to underlying conditions such as heart failure, malnutrition, or frailty.^38^, ^39^ These factors, combined with arterial stiffness and impaired autoregulation, could explain the observed U‐ or J‐curve relationships between BP and adverse cardiovascular outcomes in older populations. Our findings are therefore consistent with previous studies in similar high‐risk populations rather than contradictory to existing guidelines.

Evidence regarding BP targets in older people with T2D or CKD is complex and controversial. Existing clinical guidelines are not clear on BP targets for older people with co‐existing T2D and CKD. The UK National Institute for Health and Care Excellence (NICE) suggests reducing clinic BP to below 150/90 for adults over 80 years while emphasising clinical judgement for those with frailty or multimorbidity.^24^ The 2020 U.S. Department of Veterans Affairs and Department of Defence (VA/DoD) guidelines recommend a SBP target of <150 for individuals with diabetes aged 60 and above.^40^ The 2024 ESC Guidelines for the management of elevated blood pressure and hypertension recommend that very old and frail patients with hypertension should not be denied the potential benefits of BP‐lowering treatment targeted at 120–129/70–79, with personalised decision‐making to be considered.^15^ Additionally, the 2021 Joint Association of British Clinical Diabetologists and UK Kidney Association guideline recommends aiming for BP levels consistently below 130/80 in individuals with T2D, CKD, and albuminuria (ACR >3 mg/mmol).^17^ Among 34 hypertension guidelines, 18 recommended 150 as the systolic goal in frail and/or older patients, while four endorsed systolic targets of <130 or <120.^22^ The 2021 KDIGO Clinical Practice Guideline for the Management of Blood Pressure in CKD recommends treating adults with CKD, including those with diabetes, to a target SBP of <120 mmHg if tolerated, using standardised office BP measurement.^23^ However, KDIGO acknowledges that the evidence is limited for frail, older adults and recommends individualised treatment. These variations underscore the complexity of BP management in older adults with T2D and CKD. Our findings, which suggest that lower SBP (e.g., 120 mmHg) may be associated with higher risks of MACE and mortality than SBP around 140 mmHg, support a more conservative approach to BP targets in older adults with T2D and CKD. This aligns with the more cautious thresholds recommended by NICE and the VA/DoD guidelines. In contrast, our results suggest that the lower SBP targets recommended by some ESC and KDIGO guidance may not be appropriate for all older, multimorbid individuals, highlighting the importance of individualised treatment decisions based on frailty, comorbidities, and risk–benefit trade‐offs. SBP emerged as a stronger risk indicator than DBP in predicting MACE and mortality, which reflects established clinical understanding that systolic hypertension plays a more dominant role in cardiovascular risk.^41^, ^42^ This finding is consistent with prior studies and current risk prediction algorithms that prioritise SBP in risk stratification and treatment targets.^43^, ^44^ Nevertheless, DBP should not be overlooked, as it independently contributes to cardiovascular risk^41^ and is an integral part of hypertension diagnosis and management. Thus, comprehensive BP control should address both components to optimise outcomes in this high‐risk population. Furthermore, our findings highlight potential sex differences, which suggest that BP targets and treatment strategies should consider sex‐specific factors. Biological differences between men and women, including variations in vascular ageing, hormonal influences, and differential progression of CKD,^45^, ^46^ may partly explain the higher risks observed in men. In addition, differences in healthcare utilisation patterns, with women often more likely to engage in preventive care and monitoring, may contribute to disparities in outcomes.^47^, ^48^ A tailored approach, balancing the risks of cardiovascular events with overtreatment, is necessary. Future research should explore gaps to refine clinical recommendations improving patient care.

A key strength of this study is the use of real‐world clinical data from CPRD, providing a large, representative sample of older adults with T2D and CKD in routine clinical practice. This enhances the generalizability of our findings to similar populations in primary care settings. Additionally, the use of flexible parametric and competing risk models enhanced the robustness of our estimates. However, several limitations should be acknowledged. First, as an observational study, residual confounding cannot be ruled out despite comprehensive adjustment for key covariates. In particular, unmeasured or incompletely recorded variables such as additional comorbidities, detailed treatment status (e.g., dose and adherence of antihypertensives), and albumin‐to‐creatinine ratio (ACR) could bias the observed associations. While baseline CVD and diabetes medication use were included, a substantial proportion of participants lacked complete records, and ACR data were insufficient for inclusion. These gaps may have introduced residual confounding that could not be fully addressed. Second, BP was assessed only at a single baseline time point, which does not account for visit‐to‐visit variability over time. BP variability is clinically relevant and may influence cardiovascular outcomes, meaning that our findings could underestimate or overestimate true associations. However, our research objectives were to identify optimal baseline BP targets for cardiovascular and mortality benefit and to assess the impact of missing BP records on adverse outcomes; for these aims, the use of a single baseline BP measure was the most suitable and feasible approach given the available data. Furthermore, serial BP measurements were either not consistently recorded or were too infrequent or unreliable in our dataset to be used meaningfully. Additionally, orthostatic hypotension is common in this population, but we were unable to account for this due to a lack of relevant data. No standard measurement protocol was applied across clinics, and variation in BP assessment tools and techniques may have introduced measurement bias. Moreover, only around 25% of patients had a recorded BP measurement within 2 years prior to diagnosis, limiting assessment of BP monitoring frequency or longitudinal patterns. Future studies with access to structured orthostatic BP measurements and standardised protocols are needed to better capture these dynamics. Third, the study lacked data on ACR, an important marker of kidney damage, which limits the ability to fully characterise CKD severity. Although ACR >30 mg/g is widely used to guide more intensive therapeutic strategies,^49^ ACR data were not complete enough in our cohort to include meaningfully. When available, ACR is highly correlated with CKD stage, which we used as the primary measure of CKD severity and was the most appropriate and feasible indicator for these analyses. Future studies incorporating both ACR and CKD stage could provide a more comprehensive understanding of kidney disease severity and its interaction with BP management and outcomes. Fourth, a substantial proportion of participants had no records of diabetes or CVD medications, which may reflect under‐recording or poor treatment adherence and could have influenced the observed associations. Fifth, we were unable to distinguish between individuals with elevated BP who were receiving antihypertensive treatment and those who were untreated. While baseline CVD medication use was included as a covariate, this limitation restricts interpretation of the effect of BP control. Sixth, while we analysed BP as both a continuous and categorical variable, further stratification of categorical BP was avoided due to concerns about overinterpretation, given known variability and inaccuracies in real‐world BP recordings. Additionally, the observed lower or similar risk in individuals with high BP may reflect treatment effects and greater clinical monitoring, which could not be fully accounted for and may have introduced residual confounding. Seventh, we lacked sufficient data on baseline HbA1c levels, a marker of glycaemic control in individuals with T2D. Though potentially informative, HbA1c is often highly correlated with CKD stage,^50^ which was included in the analysis as a key covariate and was the most appropriate available proxy for disease severity in this context. Eighth, we did not have data on patient frailty, which is rarely recorded in primary care datasets and lacks a validated proxy for this population. Consequently, we cannot exclude the possibility that individuals with lower SBP (<120 mmHg) had underlying conditions such as heart failure, liver failure, or dementia, which may have contributed to their higher risks of MACE and mortality. Ninth, participants with CKD stage 5 constituted less than 1% of our cohort, and therefore the generalizability of our findings to stage 5 CKD patients may be limited. Future studies specifically designed to include sufficient numbers of stage 5 patients are needed to validate our results in this subgroup. Lastly, missing BP data may be indicative of broader healthcare access or quality issues rather than a direct effect of BP monitoring alone. This could also reflect unmeasured markers of healthcare utilisation or patient frailty, which are often not reliably captured in routine clinical databases. Although we recognise the value of exploring these factors through sensitivity analyses, our dataset did not contain suitable proxy indicators to enable such analyses. Future studies incorporating validated frailty measures and healthcare utilisation metrics would help clarify the mechanisms underlying the associations observed with missing BP data. Finally, as this was an observational study, causality cannot be inferred from the associations observed. These findings should therefore be interpreted with caution and validated in prospective studies, preferably RCTs.

## 5 CONCLUSION

This study provides novel insights into the relationships between BP levels and adverse cardiovascular risk in older adults with T2D and CKD. While lower SBP and DBP were moderately associated with higher risks of MACE and mortality, the strongest indicator of adverse outcomes was the absence of regular blood pressure monitoring. These findings emphasise the need for routine BP assessments and personalised treatment strategies to optimise cardiovascular outcomes in this vulnerable population.

## CONFLICT OF INTEREST STATEMENT

Kamlesh Khunti has acted as a consultant, speaker, or received grants for investigator‐initiated studies for Astra Zeneca, Bayer, Novo Nordisk, Sanofi‐Aventis, Servier, Lilly, Merck Sharp & Dohme, Boehringer Ingelheim, Oramed Pharmaceuticals, Pfizer, Roche, Daiichi‐Sankyo, Applied Therapeutics, Embecta, and Nestle Health Science. Francesco Zaccardi: Consultancy for Daiichi‐Sankyo, Servier, Menarini.

## Supporting information


**Data S1.** Supporting Information.

## Data Availability

CPRD's Research Data Governance (RDG) Process restricts the sharing of the original data used in this study and therefore these data are not available to share (CPRD Protocol #23_002560).
