# Supplementary material for: Blood pressure and adverse cardiovascular outcomes in older people with type 2 diabetes and chronic kidney disease: Findings based on the Clinical Practice Research Datalink databases in England
Source: Diabetes Obes Metab. 2025 Oct 28;28(1):593–605. doi: 10.1111/dom.70234 (PMC12673439; doi:10.1111/dom.70234)
Supplement: Supplementary file 1 — Data S1. Supporting Information. [file DOM-28-593-s001.docx]

Supplement 2

# Section A: CKD stage

## Figure S1: Cumulative incidence of MACE by systolic blood pressure - Adjusted and stratified for CKD stage


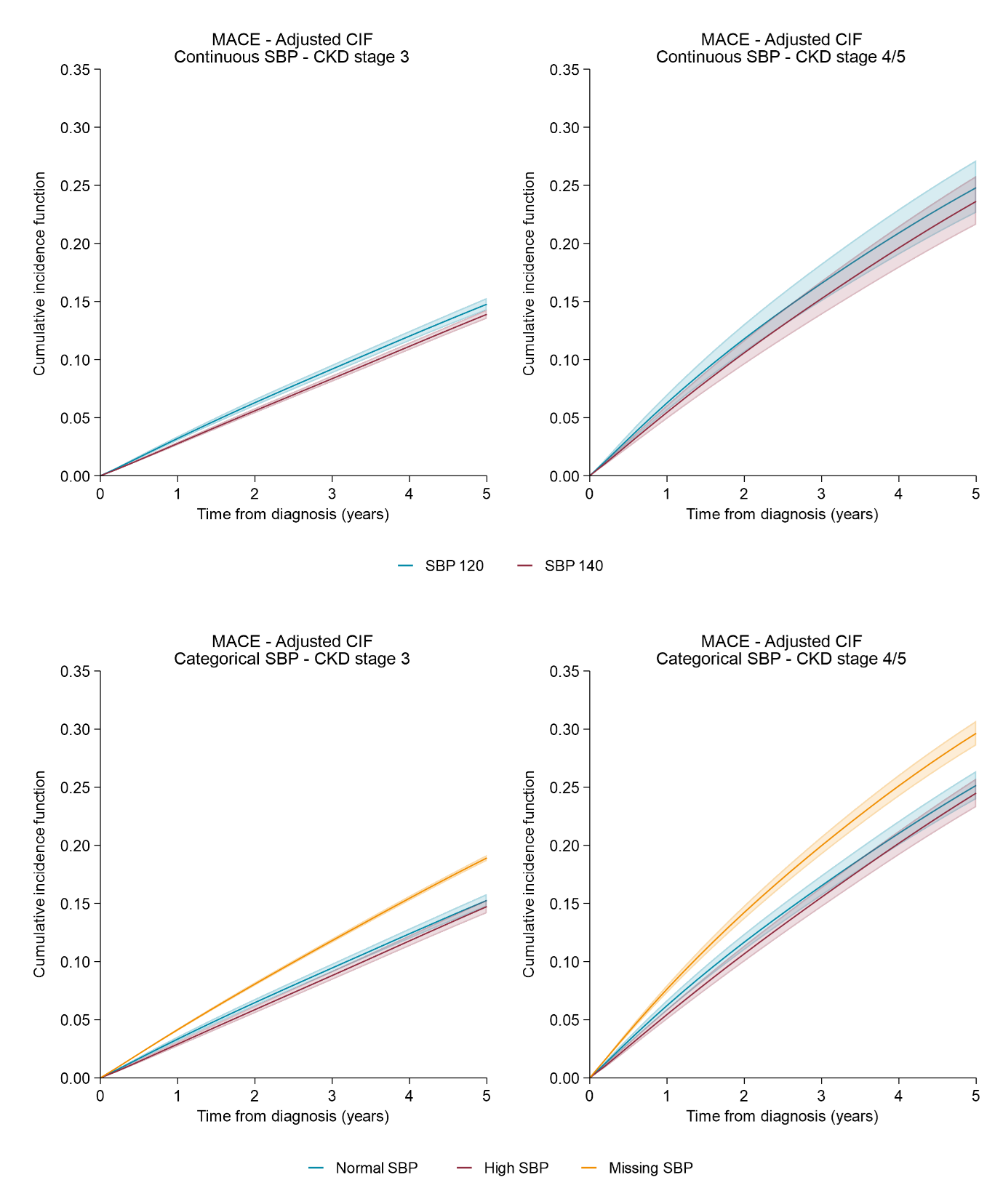


*CKD – chronic kidney disease, MACE – major adverse cardiovascular events, SBP – systolic blood pressure*

## Figure S2: Cumulative incidence of MACE by diastolic blood pressure - Adjusted and stratified for CKD stage


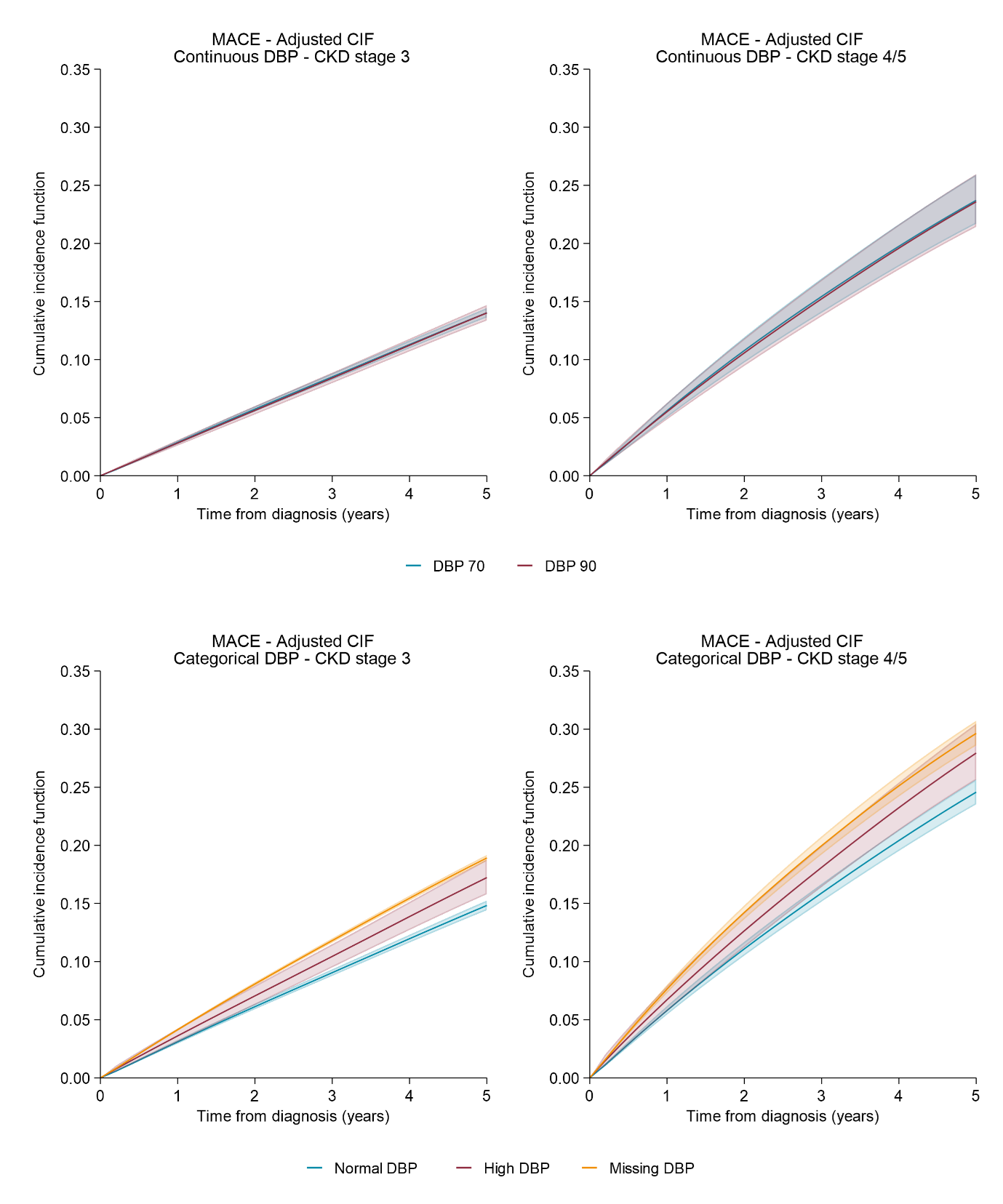


*CKD – chronic kidney disease, DBP – diastolic blood pressure, MACE – major adverse cardiovascular events*

## Figure S3: Cumulative incidence of mortality by systolic blood pressure - Adjusted and stratified for CKD stage


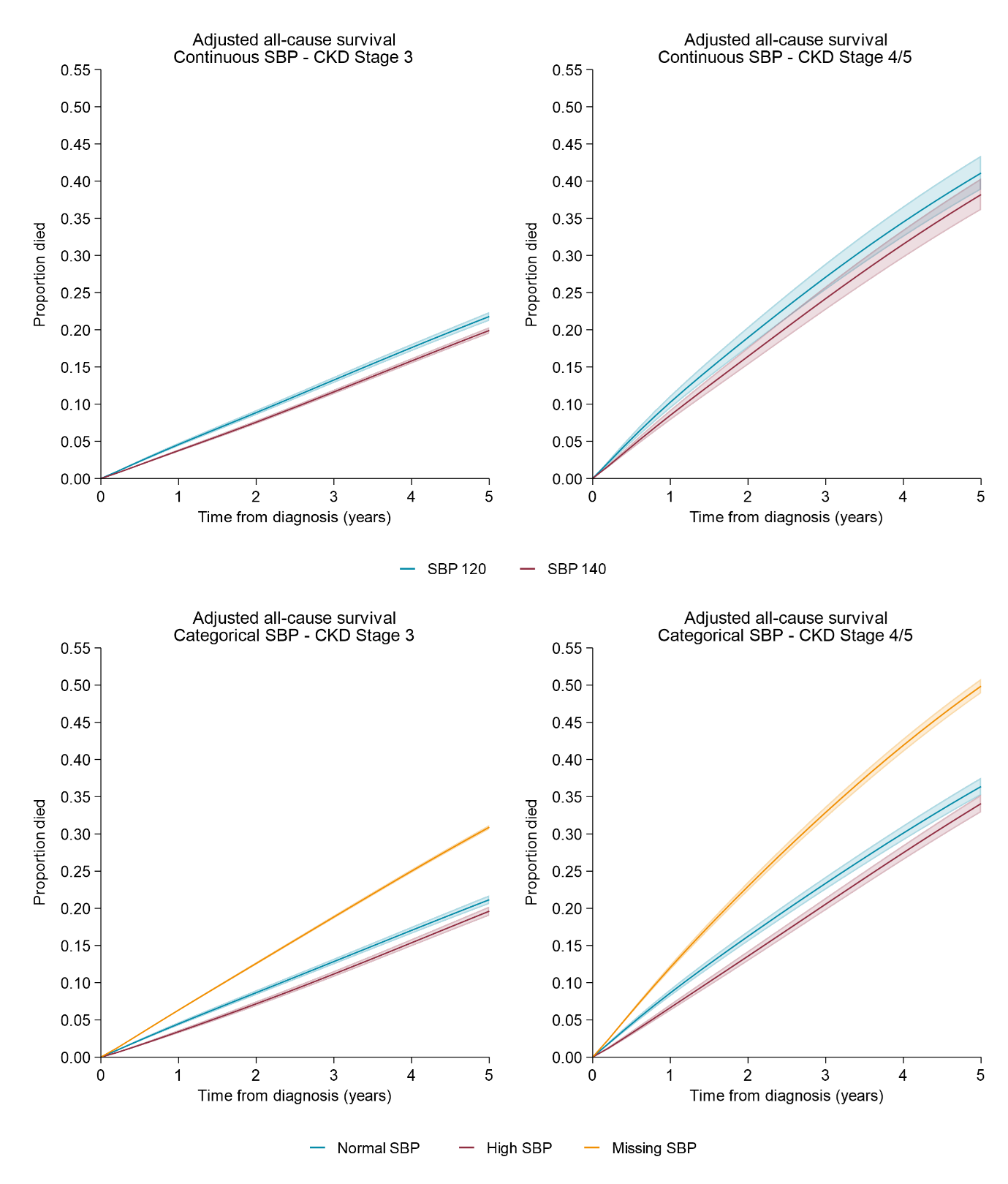


*CKD – chronic kidney disease, SBP – systolic blood pressure*

## Figure S4: Cumulative incidence of mortality by diastolic blood pressure - Adjusted and stratified for CKD stage


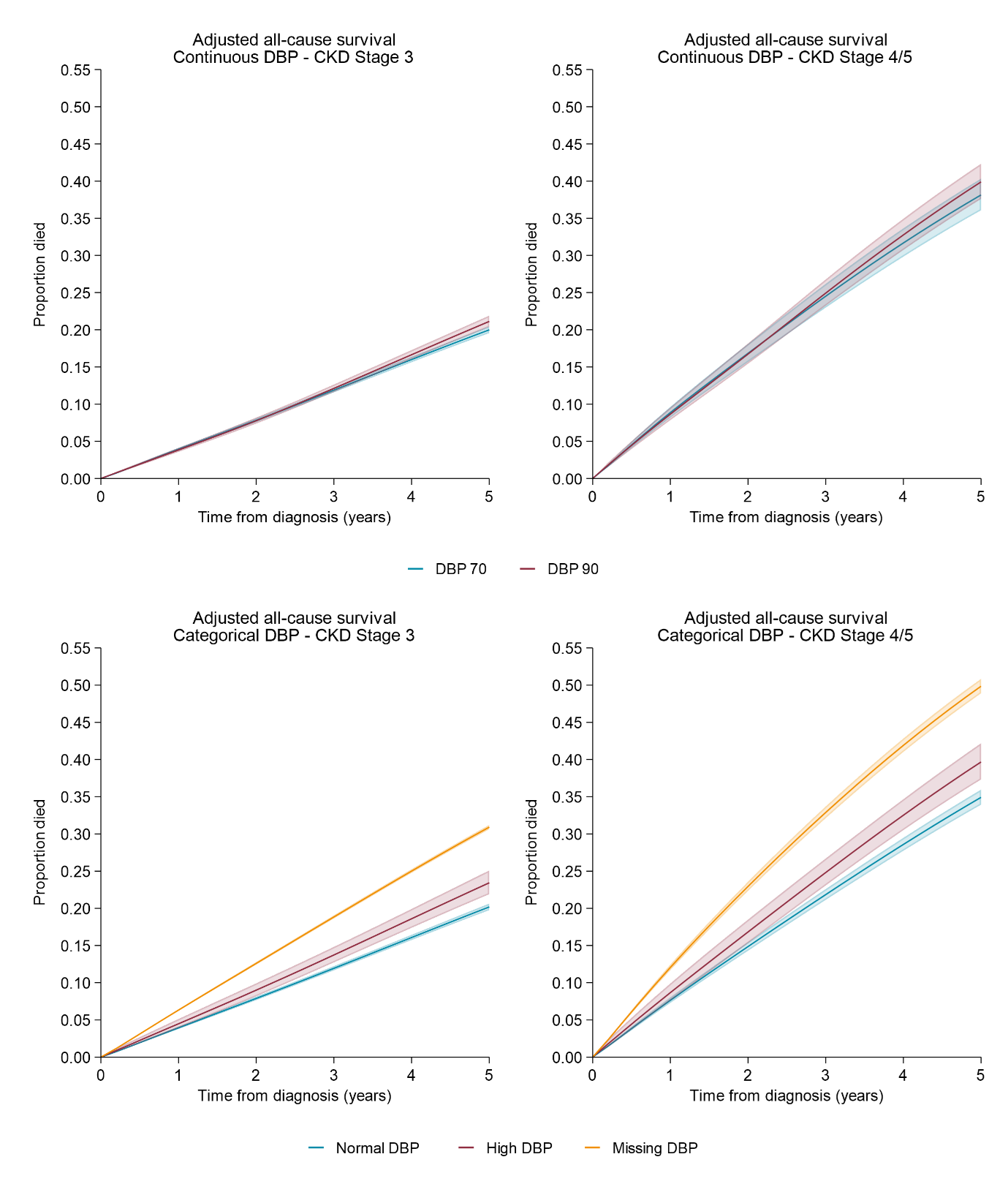


*CKD – chronic kidney disease, DBP – diastolic blood pressure*

# Section B: Cox regression

## Table S1: Adjusted Cox regression model output – MACE

| Variable | Hazard ratio (95% CI) | P-value |
| --- | --- | --- |
| Baseline SBP | 0.998 (0.996, 0.999) | 0.003 |
| Age | 1.054 (1.049, 1.058) | < 0.001 |
| Sex |  |  |
| Female | 0.740 (0.697, 0.787) | < 0.001 |
| Ethnicity |  |  |
| South Asian | 0.726 (0.610, 0.863) | < 0.001 |
| Black | 0.817 (0.662, 1.008) | 0.059 |
| Mixed | 0.734 (0.442, 1.220) | 0.233 |
| Other | 0.578 (0.429, 0.778) | < 0.001 |
| Unknown | 1.301 (1.063, 1.594) | 0.011 |
| Alcohol |  |  |
| Drinker | 0.740 (0.685, 0.800) | < 0.001 |
| Ex-drinker | 1.267 (1.056, 1.507) | 0.010 |
| Unknown | 1.160 (1.031, 1.304) | 0.013 |
| Smoking |  |  |
| Smoker | 1.134 (1.043, 1.233) | 0.003 |
| Ex-smoker | 1.255 (1.175, 1.339) | < 0.001 |
| Unknown | 0.845 (0.417, 1.711) | 0.640 |
| BMI |  |  |
| Normal weight | 0.823 (0.616, 1.101) | 0.190 |
| Overweight | 0.729 (0.546, 0.973) | 0.032 |
| Obese | 0.677 (0.507, 0.904) | 0.008 |
| Unknown | 1.700 (1.172, 2.466) | 0.005 |
| CKD stage |  |  |
| 4 | 2.196 (1.961, 2.458) | < 0.001 |
| 5 | 1.676 (1.091, 2.577) | 0.019 |
| Diabetes medication |  |  |
| Yes | 0.942 (0.885, 1.002) | 0.059 |
| CVD medication |  |  |
| Yes | 0.770(0.711, 0.833) | < 0.001 |

*Age and baseline SBP treated as continuous covariates, all others as categorical.*

*BMI – body mass index, CI – confidence interval, CKD – chronic kidney disease, CVD – cardiovascular disease, MACE – major adverse cardiovascular events, SBP – systolic blood pressure*

## Table S2: Test of proportional hazards assumption – MACE

| Variable | P-value |
| --- | --- |
| Baseline SBP | < 0.001 |
| Age | 0.278 |
| Sex |  |
| Female | 0.922 |
| Ethnicity |  |
| South Asian | 0.004 |
| Black | 0.269 |
| Mixed | 0.297 |
| Other | 0.385 |
| Unknown | < 0.001 |
| Alcohol |  |
| Drinker | 0.375 |
| Ex-drinker | 0.078 |
| Unknown | 0.191 |
| Smoking |  |
| Smoker | 0.007 |
| Ex-smoker | 0.882 |
| Unknown | 0.452 |
| BMI |  |
| Normal weight | 0.042 |
| Overweight | 0.033 |
| Obese | 0.009 |
| Unknown | 0.025 |
| CKD stage |  |
| 4 | < 0.001 |
| 5 | 0.285 |
| Diabetes medication |  |
| Yes | 0.390 |
| CVD medication |  |
| Yes | 0.005 |

*BMI – body mass index, CKD – chronic kidney disease, CVD – cardiovascular disease, MACE – major adverse cardiovascular events, SBP – systolic blood pressure*

## Figure S5: Schoenfeld test for proportional hazards – MACE, continuous covariates

*MACE – major adverse cardiovascular events, SBP – systolic blood pressure*

## Figure S6: Log-log test for proportional hazards – MACE, categorical covariates


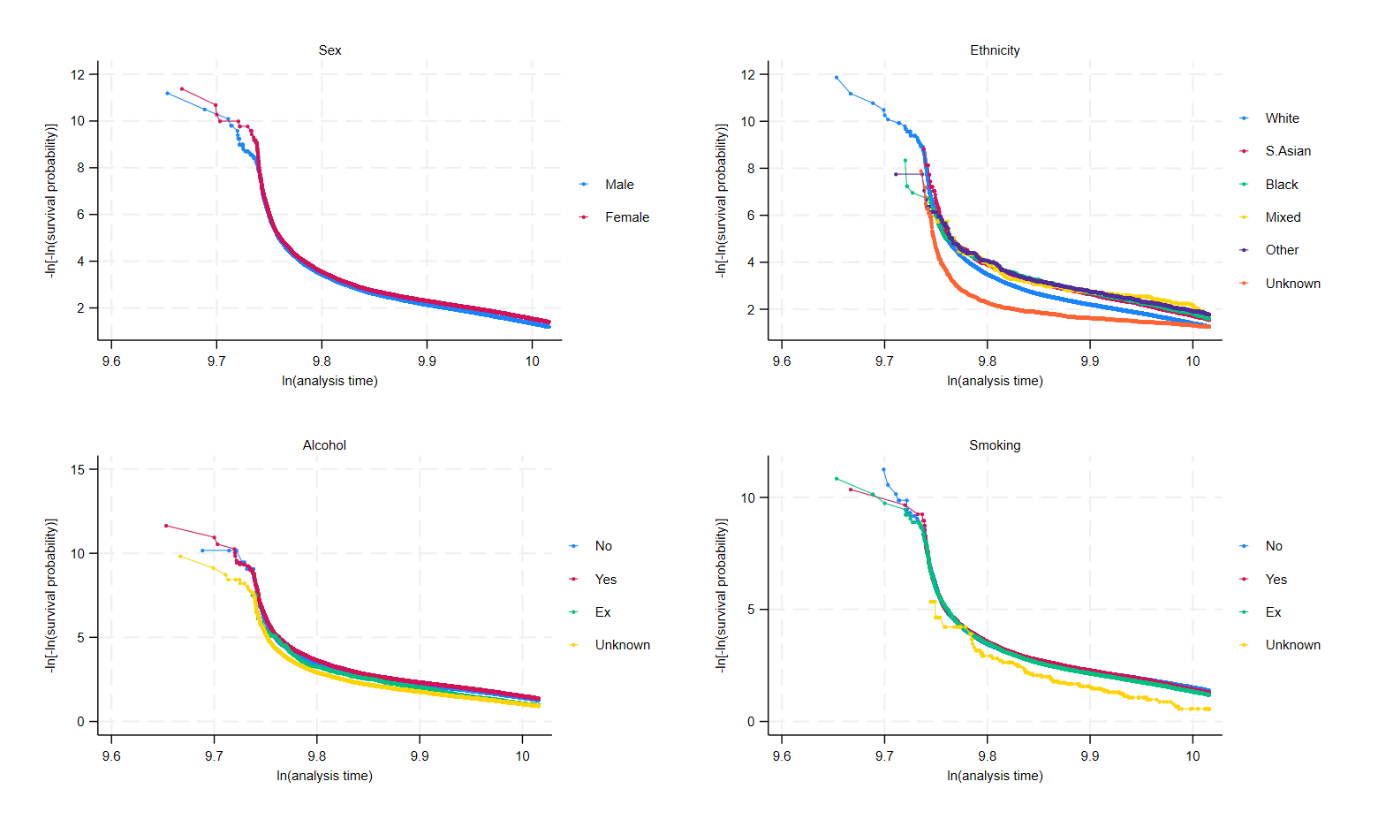

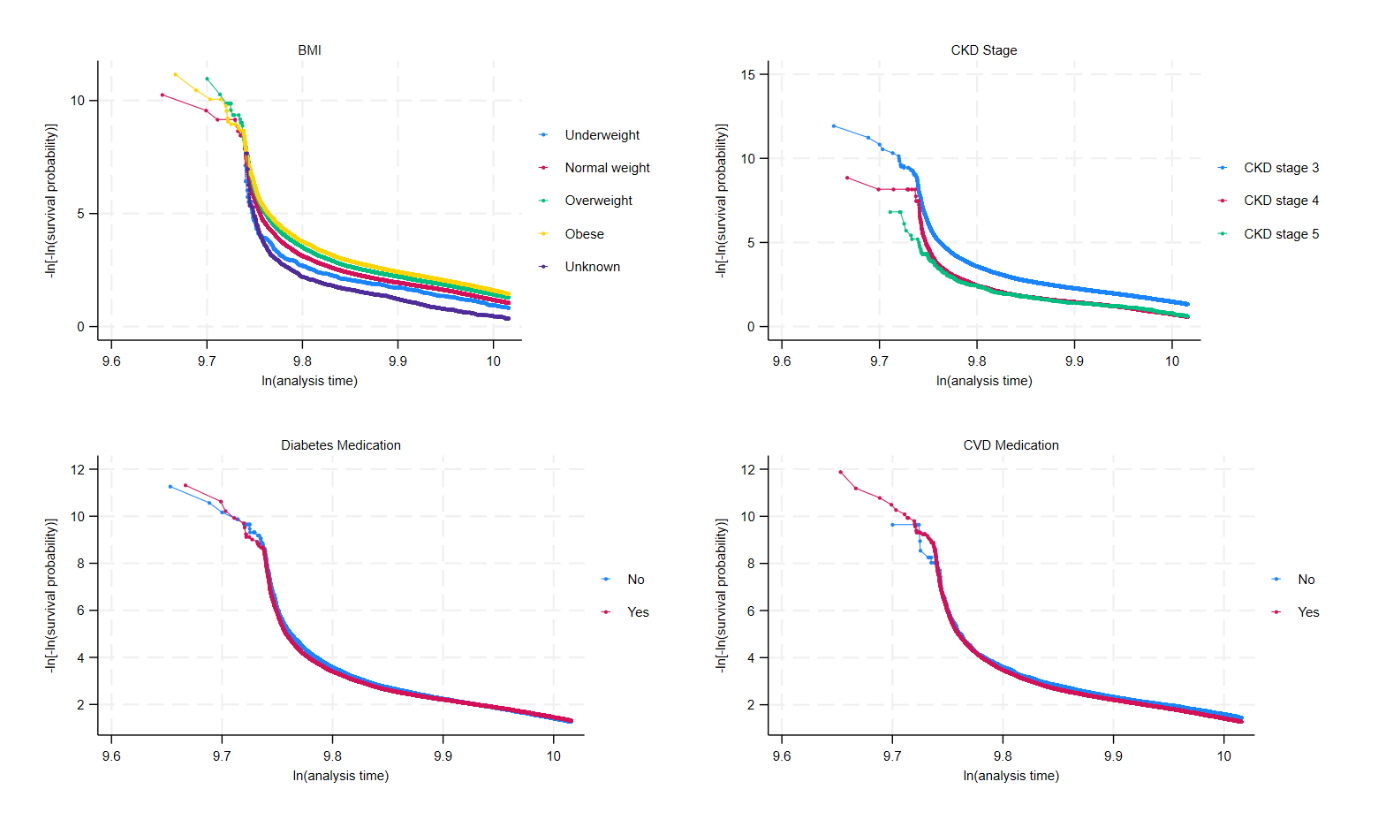


*BMI – body mass index, CKD – chronic kidney disease, CVD – cardiovascular disease, MACE – major adverse cardiovascular events*

## Table S3: Adjusted Cox regression model output – Mortality

| Variable | Hazard ratio (95% CI) | P-value |
| --- | --- | --- |
| Baseline SBP | 0.996 (0.995, 0.997) | < 0.001 |
| Age | 1.068 (1.064, 1.071) | < 0.001 |
| Sex |  |  |
| Female | 0.801 (0.761, 0.842) | < 0.001 |
| Ethnicity |  |  |
| South Asian | 0.456 (0.381, 0.546) | < 0.001 |
| Black | 0.724 (0.599, 0.875) | 0.001 |
| Mixed | 0.648 (0.412, 1.017) | 0.059 |
| Other | 0.597 (0.459, 0.760) | < 0.001 |
| Unknown | 1.700 (1.471, 1.966) | < 0.001 |
| Alcohol |  |  |
| Drinker | 0.738 (0.692, 0.786) | < 0.001 |
| Ex-drinker | 1.329 (1.157, 1.528) | < 0.001 |
| Unknown | 1.173 (1.065, 1.291) | 0.001 |
| Smoking |  |  |
| Smoker | 1.182 (1.101, 1.269) | < 0.001 |
| Ex-smoker | 1.355 (1.283, 1.430) | < 0.001 |
| Unknown | 1.064 (0.654. 1.732) | 0.803 |
| BMI |  |  |
| Normal weight | 0.619 (0.512, 0.748) | < 0.001 |
| Overweight | 0.461 (0.381, 0.557) | < 0.001 |
| Obese | 0.420 (0.348, 0.508) | < 0.001 |
| Unknown | 1.384 (1.081, 1.772) | 0.01 |
| CKD stage |  |  |
| 4 | 2.415 (2.209, 2.640) | < 0.001 |
| 5 | 3.252 (2.490, 4.248) | < 0.001 |
| Diabetes medication |  |  |
| Yes | 0.945 (0.896, 0.996) | 0.035 |
| CVD medication |  |  |
| Yes | 0.686 (0.644, 0.730) | < 0.001 |

*Age and baseline SBP treated as continuous covariates, all others as categorical.*

*BMI – body mass index, CI – confidence interval, CKD – chronic kidney disease, CVD – cardiovascular disease, SBP – systolic blood pressure*

## Table S4: Test of proportional hazards assumption - Mortality

| Variable | P-value |
| --- | --- |
| Baseline SBP | < 0.001 |
| Age | 0.028 |
| Sex |  |
| Female | 0.259 |
| Ethnicity |  |
| South Asian | < 0.001 |
| Black | 0.007 |
| Mixed | 0.817 |
| Other | 0.015 |
| Unknown | < 0.001 |
| Alcohol |  |
| Drinker | 0.492 |
| Ex-drinker | 0.045 |
| Unknown | 0.127 |
| Smoking |  |
| Smoker | < 0.001 |
| Ex-smoker | 0.133 |
| Unknown | 0.529 |
| BMI |  |
| Normal weight | 0.800 |
| Overweight | 0.535 |
| Obese | 0.093 |
| Unknown | 0.065 |
| CKD stage |  |
| 4 | < 0.001 |
| 5 | 0.0768 |
| Diabetes medication |  |
| Yes | 0.208 |
| CVD medication |  |
| Yes | 0.001 |

*BMI – body mass index, CKD – chronic kidney disease, CVD – cardiovascular disease, SBP – systolic blood pressure*

## Figure S5: Schoenfeld test for proportional hazards – Mortality, continuous covariates

*SBP – systolic blood pressure*

## Figure S6: Log-log test for proportional hazards – Mortality, categorical covariates


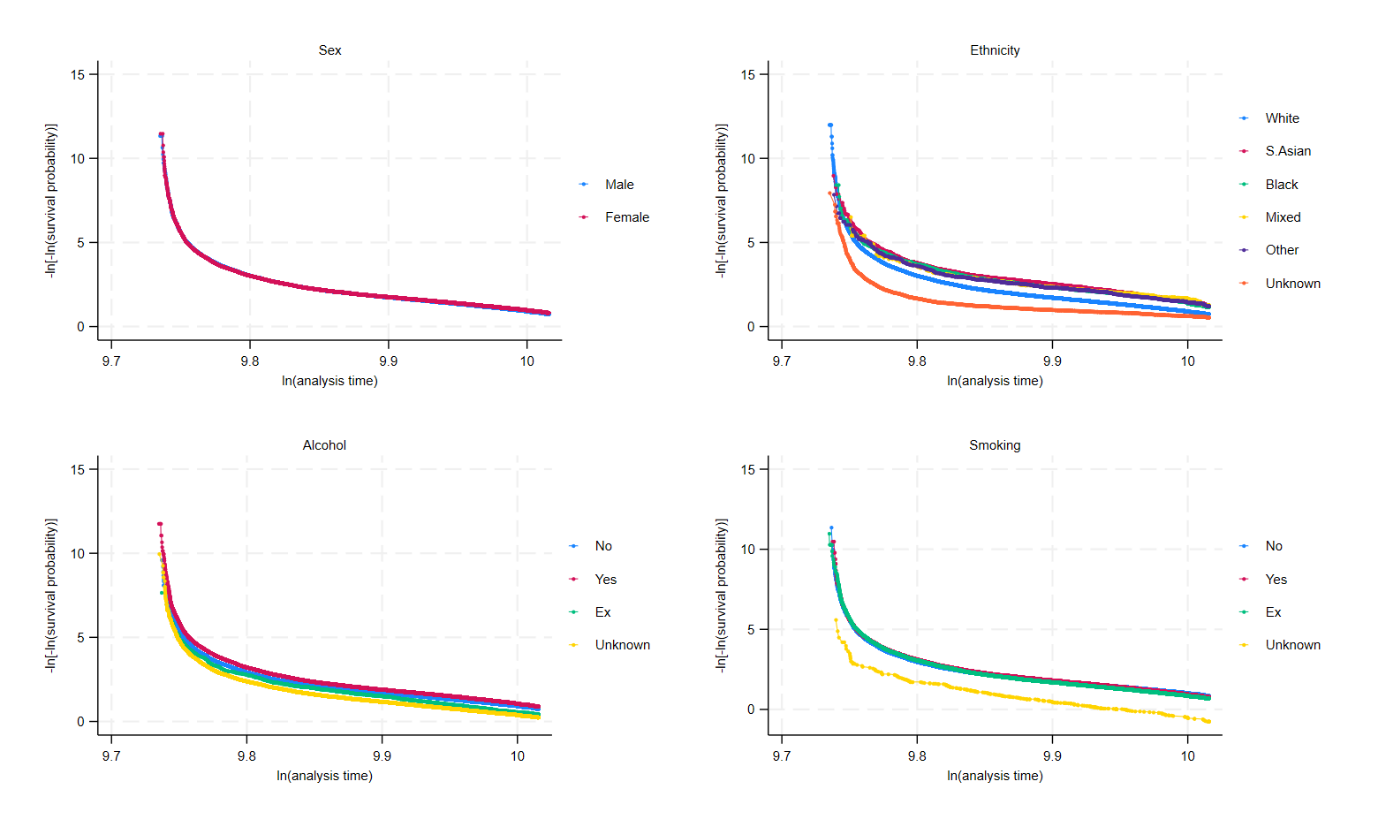

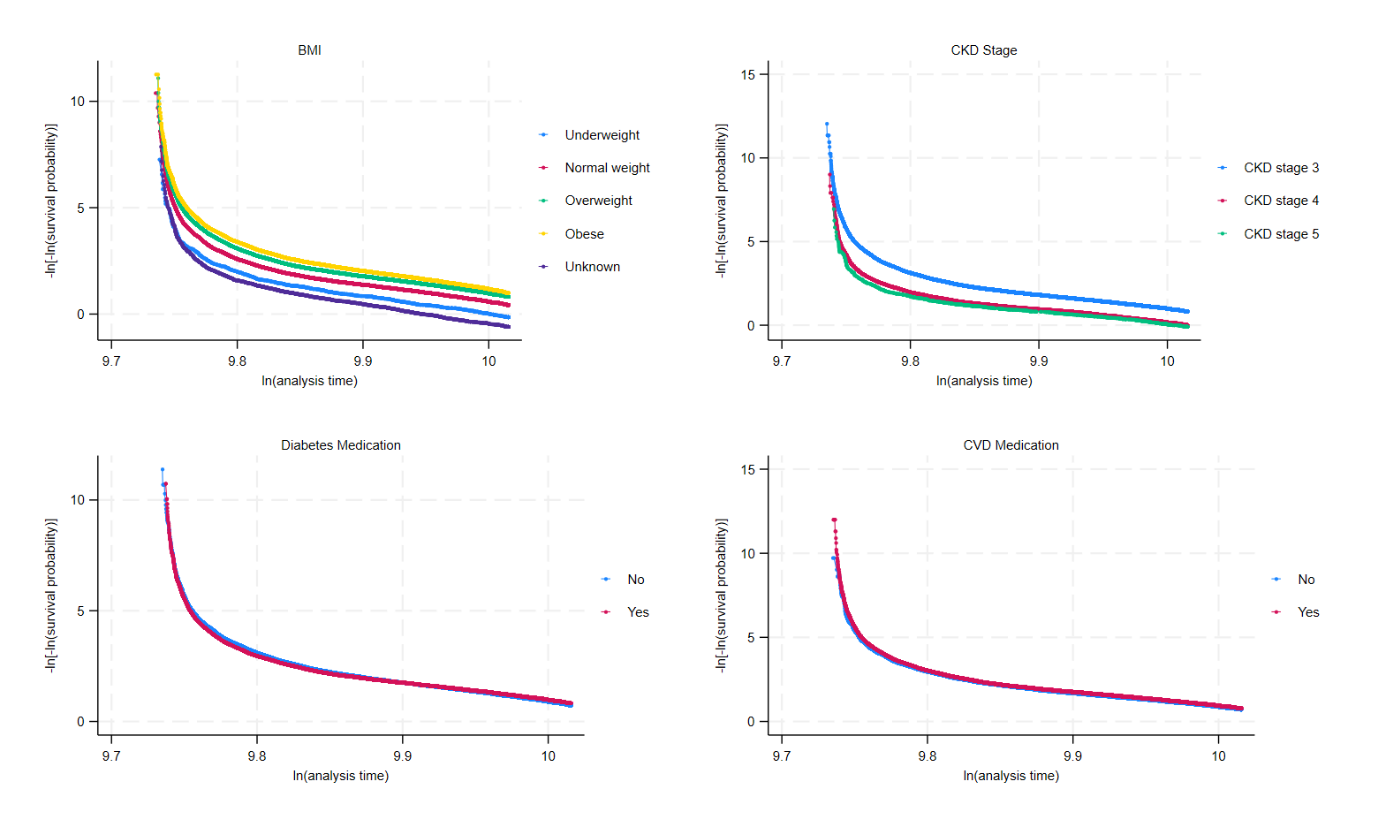


*BMI – body mass index, CKD – chronic kidney disease, CVD – cardiovascular disease*
